# Using CPAP in COVID-19 patients outside of the intensive care setting: a comparison of survival and outcomes between dialysis and non-dialysis dependent patients

**DOI:** 10.1186/s12882-021-02341-x

**Published:** 2021-04-21

**Authors:** Lauren Floyd, Madelena Stauss, Joshua Storrar, Parthvi Vanalia, Anna France, Ajay Dhaygude

**Affiliations:** 1 Department of Nephrology, Royal Preston Hospital, Lancashire Teaching Hospitals NHS Foundation Trust, Preston, UK; 2 University of Central Lancashire, Lancashire, UK

**Keywords:** CPAP, COVID-19, Dialysis, Anticoagulation

## Abstract

**Background:**

SARS-CoV-2 (COVID-19) is a novel coronavirus associated with high mortality rates. The use of Continuous Positive Airway Pressure (CPAP) has been recognised as a management option for severe COVID-19 (NHS, Specialty guides for patient management during the coronavirus pandemic Guidance for the role and use of non-invasive respiratory support in adult patients with coronavirus (confirmed or suspected), https://www.nice.org.uk/guidance/ng159). We offered ward-based CPAP to COVID-19, dialysis patients not suitable for escalation to ICU. The aim of the study was to evaluate the use of CPAP for COVID-19 dialysis patients compared to non-dialysis COVID-19 patients outside of the intensive care setting. We further aimed to investigate factors associated with improved outcomes.

**Methods:**

Data was collected from a single centre (Royal Preston Hospital, UK), from March to June 2020. Treatment outcomes were compared for dialysis and non-dialysis dependent patients who received CPAP with limitations on their escalation and resuscitation status. Kaplan-Meier survival curves and Cox regression models were used to compare outcomes. The primary study outcome was 30 day mortality. Confounders including length of admission, systemic anticoagulation and ultrafiltration volumes on dialysis were also analysed.

**Results:**

Over the study period, 40 dialysis patients tested positive for COVID-19, with 30 requiring hospital admission. 93% (*n* = 28) required supplementary oxygen and 12% (*n* = 9) required CPAP on the ward. These patients were compared to a serial selection of 14 non-dialysis patients treated with CPAP during the same period. Results showed a significant difference in 30 day survival rates between the two groups: 88.9% in the dialysis group vs. 21.4% in the non-dialysis group. Statistical modelling showed that anticoagulation was also an important factor and correlated with better outcomes.

**Conclusion:**

This is to the best of our knowledge, the largest series of COVID-19 dialysis patients treated with CPAP in a ward-based setting. In general, dialysis dependent patients have multiple co-morbidities including cardiovascular disease and diabetes mellitus making them vulnerable to COVID-19 and not always suitable for treatment in ICU. We showed a significantly lower 30 day mortality rate with the use of CPAP in the dialysis group (11.1%) compared to the non-dialysis group (78.6%). Despite a small sample size, we believe this study provides impetus for further work clarifying the role of CPAP in treating COVID-19 dialysis dependent patients.

**Supplementary Information:**

The online version contains supplementary material available at 10.1186/s12882-021-02341-x.

## Background

SARS-CoV-2 (COVID-19) is a novel coronavirus which has become a global pandemic in the space of a few months. The presentation, course and definitive treatment of the virus remains unclear with continually emerging evidence. Royal Preston Hospital is in the North West of England and serves a population of 1.5million, with a total of 610 dialysis patients; home haemodialysis (HHD), in-centre haemodialysis (ICHD) and peritoneal dialysis (PD). Following the trajectory of London’s dialysis population experience, which was roughly 3–4 weeks ahead of the North West, we anticipated a worst case scenario of 20–22% of our dialysis patients testing positive for COVID-19 [1]. Based on this assumption, the number of patients (~ 120) in our region potentially requiring hospital admission and further treatment such as Continuous Positive Airway Pressure (CPAP) would have exceeded the capacity of our centre’s intensive care unit (ICU) by two-fold. With this projection and compounded by the frailty and multimorbid nature of our dialysis patients, we looked at delivering CPAP as a ward-based treatment. Medical and nursing teams, including dialysis staff received education from the respiratory department in how to effectively set up, commence and manage CPAP.

The role of CPAP was recognised early on as a management option in severe COVID-19 but its efficacy remains unknown. CPAP is a form of respiratory support that can be used to help patients with respiratory distress, type 1 respiratory failure and increasing oxygen requirements. It has been recognised as important in the management of COVID-19 patients and was advised by NHS England early in the pandemic [2]. The role of CPAP in the COVID-19 pandemic is 2 fold; one as a treatment option for those not suitable for intubation and ventilation, and the second as a method of relieving unprecedented pressure on ICU’s [3]. The use of CPAP in the intensive care setting has been used for many decades as a management option for pulmonary oedema and acute respiratory distress. However, its use as a ward-based treatment in those with limitations of care is less common and often limited to high dependency and respiratory high care settings.

Our study looked at the use of CPAP as a ceiling of care treatment in COVID-19 dialysis dependent patients who were not suitable for escalation to level 2 or 3 care, defined as patients requiring greater levels of observation and intervention or those needing advanced respiratory support together with the support of at least two organ systems [4]. Offering CPAP for dialysis patients in a renal ward was a novel service development we implemented due to limited access to ICU beds. We were able to successfully deliver this new service with rapid training of all ward-based staff in a very short period of time. The objectives of the study were to describe the outcomes for COVID-19 dialysis patients compared to non-dialysis COVID-19 patients treated with CPAP outside the intensive care setting and to further investigate factors associated with improved outcomes.

## Methods

The patients included in this study were dialysis dependent (HD, ICHD, PD) with COVID-19 who required respiratory support in the form of CPAP. All patients included received CPAP, two of whom were already on nocturnal CPAP for obstructive sleep apnoea at home but required escalation to 24 h respiratory support. CPAP was delivered on the renal ward with educational and clinical support from the respiratory department. Dialysis was offered simultaneously with CPAP and patients were nursed in cohorted bays with full Personal Protective Equipment (PPE).

Single centre, real time data collection occurred from 24th March until 1st June 2020 (the peak incidence in our region). The following data was collected: patient demographics, clinical frailty scores (CFS), escalation status, length of stay, indications for CPAP as well as frequency of dialysis and ultrafiltration volumes. Primary outcome measures were overall patient survival and 30 day mortality. Investigations were reviewed including laboratory parameters, imaging and the use of procalcitonin as a marker of bacterial infection. We also measured outcomes looking at tolerance and duration of CPAP as well as length of hospital admission.

Fourteen non dialysis patients were included in this study. The patients included were consecutive patients admitted to the respiratory medical ward during the peak study period who required CPAP, but who were not felt to be suitable for escalation to level 3 care. This was based on clinical assessments made by the medical team on admission that took into account the degree of co-morbidities and clinical frailty score, which included assessment of patient’s functional baseline.

Univariate summaries were used to explore the data. With regards to demographics and comorbidities, cross tabulations of each variable against dialysis group were used to examine the differences between the dialysis and non-dialysis groups. Kaplan-Meier survival curves were used to explore factors related to 30 day mortality and Cox proportional hazards modelling was used to assess the significance of these factors for survival. All statistical analyses were conducted in R version 3.6.1. Ethics approval and consent to participate were not applicable to this study.

## Results

Over the study period, 40 dialysis patients tested positive for COVID-19, with 30 requiring admission to hospital for COVID-19 related illness. Of these 30 patients, 93% (*n* = 28) required supplementary oxygen therapy and 12% (*n* = 9) deteriorated requiring treatment with CPAP on the ward. Of those that received CPAP the average CFS was 5, which was defined as people who have evidence of slowing, needing help with activities of daily living (ADL’s) and are unable to walk outside alone [5]. The dialysis cohort were compared with 14 general medical patients who were not dialysis dependent but were co-morbid (Table 1) and not felt to be suitable for escalation to level 3 care.

**Table 1:** Comparisons in demographics, co-morbidities and primary outcomes across the study and between the non dialysis and dialysis groups

	Total (*n* = 23)	Dialysis Dependent (*n* = 9)	Non-Dialysis (*n* = 14)
Demographics			
Age (years),_*Median (IQR)*_	70 (66–78)	70 (53–71)	71.5 (66–80)
Sex			
Male, _*n (%)*_	15 (65.3)	7 (77.8)	8 (57.1)
Female, _*n (%)*_	8 (34.7)	2 (22.2)	6 (42.9)
Clinical Frailty Score, _*Median*_	4.7	5.4	4.3
Disease Burden			
eGFR (ml/min), _*Median*_			64
Procalcitonin (ng/ml),_*Median*_	1.58	3.89	0.43
Hx chronic lung disease, _*n (%)*_	6 (26.1)	2 (22.2)	4 (28.6)
Hx ischemic heart disease, _*n (%)*_	18 (78.2)	7 (77.8)	11 (78.6)
Primary outcomes			
30 day mortality, _*n (%)*_	12 (56.5)	1 (11.1)	11 (78.6)

Dialysis patients had a higher proportion of males compared to the non-dialysis group (77.8% vs 57.1% respectively). Clinical frailty scores (5 vs 4) were similar between the dialysis and non-dialysis groups respectively (Table 1). Considering age, while the median ages differed by only 1.5 years, the interquartile range (IQR) showed a generally older age distribution in those not receiving dialysis (Table 1). The incidence of chronic lung disease and ischemic heart disease was similarly proportioned between the dialysis and non-dialysis groups. The non-dialysis patients had a median eGFR of 64 ml/min with five patients having an eGFR of > 90 ml/min on admission, meaning this cohort was predominantly free from CKD. CKD is considered as a risk factor for poor outcomes in COVID-19 [6].

Exploratory analysis using univariate Cox regression, modelling each variable separately for survival, did not detect a significant difference in gender or history of chronic lung disease and ischemic heart disease on 30 day survival for the sample as a whole. Furthermore, whilst not statistically significant, univariate Cox regression demonstrated a poorer prognosis in older patients over 80 years, whilst those under 60 years had an increased chance of survival. We noted that, all patients aged over 80 were in the non-dialysis group and all those under 60 were in the dialysis group within this sample. Although not validated in large populations, a ‘COVID age’ can be calculated based on patients risk factors including age, gender, ethnicity and co-morbidities [7]. Evidence suggests a significantly higher ‘COVID age’ is associated with end stage renal disease and increased mortality. For example a calculated ‘COVID age’ of a 60 year old, Caucasian dialysis patient would be 83 years [7, 8]. When calculating the ‘COVID age’ of our cohorts, the dialysis patients had a markedly higher average ‘COVID age’ of 110.7 years compared to the non-dialysis group (86 years).

All patients had a positive viral PCR nasopharyngeal swab for SARS-CoV-2 and received CPAP in a ward-based setting. Two (22.2%) patients were discussed with critical care regarding suitability for escalation and only one patient was admitted for CPAP in the ICU setting, where they were initiated on CPAP and later stepped down for ongoing CPAP on the ward. No patients were considered for intubation and ventilation or cardiopulmonary resuscitation across both groups. All patients had a documented ceiling of care decision made, with 89% having this decision made prior to commencing CPAP. Seventy seven percent had evidence of COVID-19 related chest x-ray changes at the time of starting CPAP. Two patients in the renal dialysis group and three in the non-dialysis group started CPAP but failed to tolerate the therapy and withdrew from CPAP within 48 h.

Patients initiated on CPAP met the criteria based on tachypnoea with a respiratory rate of over 25 breaths per minute along with oxygen requirements of over 40% inspired oxygen (FiO2 0.4). Across the dialysis and non-dialysis groups, the median time spent on CPAP was 6 days and 5.5 days respectively, with one dialysis dependent patient requiring 15 days of respiratory support. CPAP settings were similar across both cohorts, most patients required a maximum FiO2 of 0.6 (60% inspired oxygen) and the average Positive End Expiratory Pressure (PEEP) achieved was 12 cmH_2_O, with one patient requiring a PEEP of 16 cmH_2_O.

**Fig. 1:**
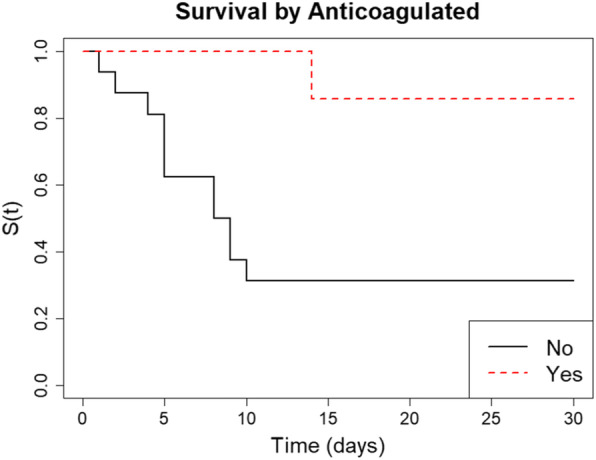
Kaplan-Meier survival curves showing the probability of survival up to 30 days for COVID-19 positive CPAP patients, stratified by dialysis group

Analysis of the effect of dialysis on the primary outcome using Cox regression detected a statistically significant difference in 30 day survival rate (*p* = 0.012). Thirty day survival was 88.9% in the dialysis group and 21.4% in the non-dialysis group, and the univariate Cox regression model (Table 2) suggested being on dialysis was associated with a 93% decrease in hazard of 30 day mortality compared to not being on dialysis *(*Fig. 1*).* The average length of stay in the dialysis group was much higher at 26.5 days compared to the non-dialysis group which was 12.2 days. This is likely to be explained by the difference in mortality data and reflects that many of the non-dialysis patients had died by day 10 of admission (Fig. 1).

**Table 2:** Results of univariate Cox regression modelling, showing hazard ratios (HR), 95% confidence intervals (CI) and *p*-values

Variable	HR (95% CI)	***p***-value
**Dialysis** (No)		
Yes	^a^0.071 (0.009,0.560)	0.012
**Age (Years)** (0–60)		
61–70	0.288 (0.336,24.65)	0.335
71–80	2.258 (0.234,21.75)	0.481
81 +	7.600 (0.768,75.15)	0.083
**Age (Years)**	1.050 (0.987,1.117)	0.119
**Sex** (Female)		
Male	0.511 (0.161,1.626)	0.256
**CPAP Days**	0.896 (0.775,1.035)	0.135
**Max PEEP**	0.964 (0.838,1.284)	0.734
**Anticoagulated** (No)		
Yes	^a^0.122 (0.016,0.952)	0.045

^a^Significant at 5% significance level

- Variable column bracket key: **(Units)** (Baseline reference category)

In the dialysis dependent cohort patients received standard treatment with intermittent hemodiafiltration (HDF) and high flux dialyzers. The majority of patients (78%) received daily or alternate day dialysis for a duration of 2–4 h each session. There was an average ultrafiltration volume of 9 l in their first week on CPAP. Two haemodialysis patients required fluid removal of over 18 l in the first 7 days due to clinical features of pulmonary oedema and fluid overload.

**Fig. 2:**
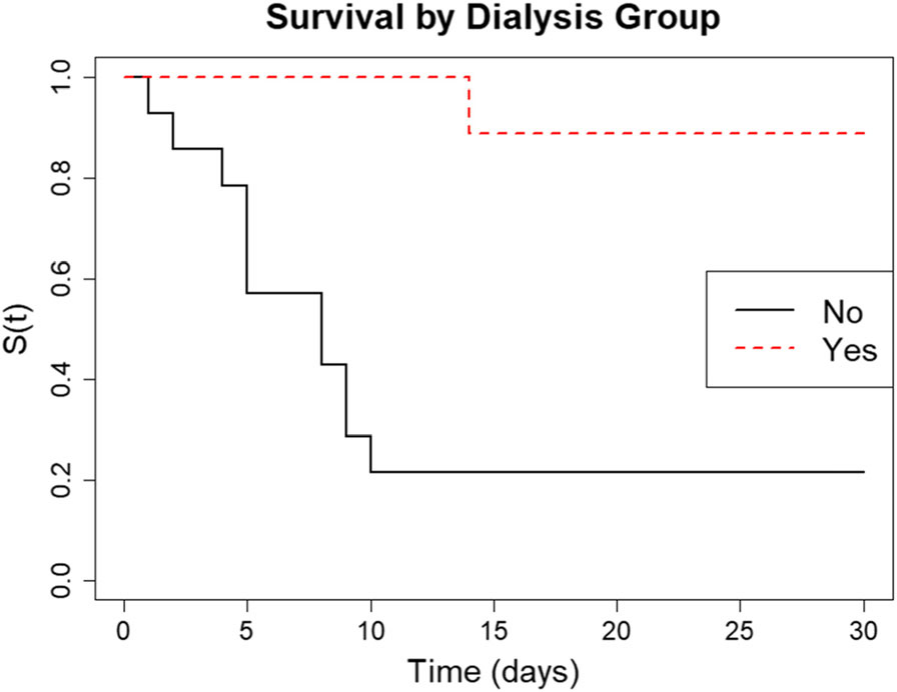
Kaplan-Meier survival curves showing the probability of survival up to 30 days for COVID-19 positive CPAP patients, stratified by anticoagulation

In the non-dialysis group only 14% (*n* = 2) were systemically anticoagulated, in contrast to 56% (*n* = 5) in the dialysis group (not including the low molecular weight heparin they received on dialysis). Kaplan-Meier survival curves show that the administration of anticoagulation results in a statistically significant difference in survival rate between the two groups (Fig. 2).

## Discussion

Our study demonstrated an improved survival in dialysis patients treated with ward-based CPAP compared to non-dialysis patients. The reasons behind the improved outcomes in our dialysis cohort are likely to be multifactorial and potentially confounded. Our study was limited by a small sample size and by our comparative group not being equally matched across all demographics and co-morbidities. The unmatched cohorts means there is possible confounding of the relationship between dialysis and the survival outcome and this would be an area for further investigation.

It is widely recognised that dialysis patients have altered immunity secondary to the chronic uraemic state, which may be compounded by immunosuppressant use [9]. Initial reports suggested dialysis patients would be at higher risk due to increased prevalence of hypertension, diabetes mellitus and cardiovascular disease, but also the need to attend dialysis units regularly; often via public transport making social distancing and isolating difficult [10–12]. One of the proposed mechanisms for Acute Respiratory Distress Syndrome (ARDS) and Multiorgan Failure (MOF) in the context of COVID-19 is the cytokine release storm (CRS) [13]. As a direct result of viral invasion into the host alveolar cell via the ACE2 receptor [14], there is a cascade of reactions resulting in widespread release of proinflammatory cytokines which include interleukins, particularly interleukin-6(IL-6), and tumour-necrosis factor alpha (TNF-α) [15]. It has been hypothesised that the immunomodulatory effect of the uraemic environment in dialysis patients tempers this cytokine response to some extent [16]. In addition to lower levels of inflammatory cytokines seen in haemodialysis patients compared to non-dialysis controls, haemodialysis patients have also been shown to have lower numbers of T cells, T helper cells, T killer cells, NK cells and B lymphocytes; with cell numbers reduced further still in haemodialysis patients with COVID-19 disease compared to those without [17]. When comparing mortality, higher levels of inflammatory markers including IL-6 were found in haemodialysis patients who died compared with survivors [18]. So, whilst on the one hand, dialysis patients are prone to increased infections in general because of their immunosuppressed state, on the other it appears to confer some protection from the harmful cytokine mediated effects of COVID-19. Indeed, it has been shown that dialysis patients have milder clinical features of COVID-19 when compared to the general population [17, 19, 20]. Whilst our study looks at those patients that had a more severe course of illness it is important to note that only those who were symptomatic were screened for COVID-19 and therefore many asymptomatic patients may have been missed, making it hard to determine the true incidence of COVID-19 in our dialysis group.

**Fig. 3:**
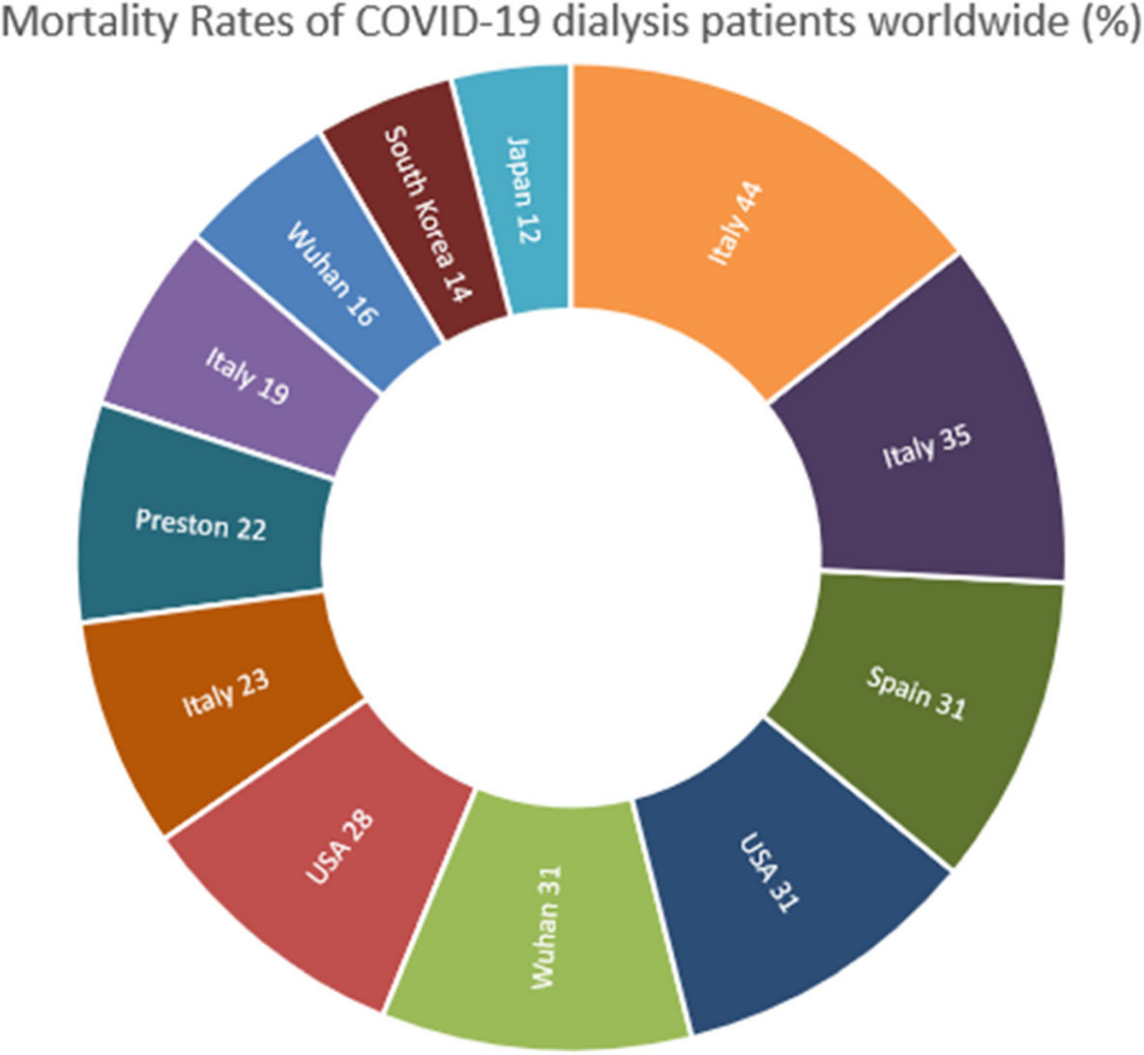
Mortality rates amongst COVID-19 dialysis patients from case reports in different centres worldwide, expressed as percentages [11, 14, 17–19, 22–27]

We report a 30 day mortality rate of 11.1%, with an overall mortality rate of 22.2% at the time of writing, within our dialysis patients that received CPAP. Various reports have shown differing mortality rates amongst the dialysis patients they investigated; ranging from no deaths [12, 21] and very mild disease [20] in the initial reports published from China, up to as high as 44% in Italy [22]. Excluding those with no mortality, Fig. 3 depicts the worldwide mortality rates across the dialysis groups, which on average are in keeping with our findings. However it is important to note the small sample size in all studies, which in addition to discrepancy in clinical practice and dialysis populations, may account for the highly variable mortality rates. Furthermore, our mortality rate refers to dialysis patients who received ward-based CPAP, a group who by definition are likely to have been more acutely unwell due to the need for respiratory support.

Within our study the mortality rate of non-dialysis patients was very high at just under 80%, which is substantially higher than the region of 5% seen in the general population worldwide [28], although this includes all COVID-19 infections and not the most severe cases needing CPAP as reflected by our cohort. In addition, our cohort of non-dialysis patients required inpatient care and were deemed unsuitable for escalation to ICU. This was due to multiple factors reflected by their comorbidities, average CFS of 4 and median age over 70. This cohort is therefore not representative of the general population, and not reflective of our centre’s overall mortality rate for COVID-19.

Regarding escalation and treatment of COVID-19 positive dialysis patients to ICU, very little is known. Assessment for admission to intensive care is challenging and more recently the use of CFS is being used to determine patients’ risk of hospital associated mortality, high CFS are associated with worse outcomes [29]. With regards to respiratory support, of the two published studies, both hailing from New York USA, the mortality rate in mechanically ventilated dialysis patients was very high at 75% [23] and 94.7% [18]. More outcome data in this area is required to drawn any firm conclusion, however if the significant mortality rate is replicated elsewhere then providing ward-based CPAP or level 2 respiratory support in a feasible way holds all the more weight.

In our cohort of dialysis patients almost all of those receiving CPAP had either daily or alternate day ultrafiltration via haemodialysis. Accumulating evidence has shown that positive fluid balance is a strong and independent predictor of poor outcomes in the critically ill. Positive fluid balance is often the result of fluid administration in acutely unwell patients in addition to maintenance fluids that are prescribed during admissions [30, 31]. The administration of fluids in non dialysis groups is often more common and can results in fluid overload and pulmonary oedema, which can lead to worse outcomes [32]. The role of significant fluid removal in our patients reflects other common practices prior to COVID-19 in a critical care setting [33]. A prospective study in 2002 showed that daily dialysis resulted in lower rates of multi organ failure and fluid imbalance compared to alternate day dialysis [34]. Specifically, in the setting of ARDS, influx of inflammatory cells and cytokines results in marked interstitial oedema and stiff lungs, for which higher volumes of ultrafiltration may have a beneficial effect. Studies reporting specific parameters for renal replacement therapy and fluid removal in dialysis patients with COVID-19 are scarce, however of those case reports where the patient survived there were large volumes of ultrafiltration, namely 8.5 l over 53 h of Continuous Veno-Venous Haemofiltration (CVVH) [21] and 7 kg reduction in body weight over five days [24]. Conversely, an early study reported no difference in disease severity related to dialysis frequency, although no ultrafiltration volumes were provided [11]. It is important to note that in our cohort we used intermittent haemodialysis in comparison to other studies where patients in the critical care setting are likely to receive continuous renal replacement therapy in the form of CVVH. The full extent of increased metabolic by product and possible inflammatory cytokine clearance is unknown between these modalities. As such, making a direct comparison between HD and CVVH in relation to outcomes amongst COVID-19 patients and critically ill dialysis patients is limited.

Within our cohort the use of anticoagulation appeared to be associated with reduced mortality, with improved survival in those patients who were systemically anticoagulated. This result was statistically significant. This is in keeping with other studies, where treatment with heparin was associated with improved survival in severe COVID-19 disease [35, 36]. Fifty-six percent of our dialysis patients compared to 14% of non-dialysis patients were systemically anticoagulated although the survival benefit may be confounded by the impact of dialysis on this group. The lower rates of systemic anticoagulation seen in the non-dialysis group likely reflect the fact that data collection occurred early in our experience of COVID-19. Our anticoagulation practice now is in keeping with published guidance. COVID-19 is now well recognised as a procoagulant state, with particular emphasis on higher levels of d-dimer and fibrin degradation products being associated with poorer outcomes [35, 36]. This was further evidenced by three of our dialysis patients who developed A-V fistula thrombosis during their COVID-19 illness, requiring alternative vascular access. Data related specifically to anticoagulation in dialysis cohorts is scarce. Within a French study of patients admitted to ICU, despite either prophylactic or therapeutic anticoagulation, 96.6% of patients receiving continuous RRT developed a clotted dialysis circuit [37]. In addition they reported increased thrombotic complications, although the impact of ultrafiltration in these groups may have been contributory [37]. Within our centre dialysis patients are routinely anticoagulated on dialysis with the low molecular weight heparin (LMWH) tinzaparin, unless there is a specific contra-indication to doing so, which may further contribute to the systemic anticoagulation they were otherwise receiving whilst admitted as an inpatient. Outside of the primary effect of preventing thrombus formation, treatment with heparin has been suggested to be beneficial inpatients with COVID-19 due to a variety of different mechanisms, including anti-inflammatory properties, mitigating and protecting against damage to the vascular endothelium, and interfering with the coagulation systems role in the pathogenesis of ARDS [38]. Furthermore, heparin has been shown to have a potential anti-viral effect in COVID-19, which may contribute to the more favourable outcomes seen in anticoagulated patients [38–40]. Given the above findings, more studies are required looking at the specific role of anticoagulation in dialysis patients and those receiving acute RRT during the COVID-19 pandemic.

## Conclusion

Whilst there are limitations to the study based on the small sample size, it is to the best of our knowledge, the largest series of dialysis dependent COVID-19 patients treated with CPAP in non ICU settings. We show a 30 day mortality rate of 11.1% which is significantly lower than that of the non-dialysis patients (78.6%). The role of ultrafiltration and anticoagulation may also be a variable that has contributed to reduced mortality rate in this cohort. There was no statistically significant difference between age and gender, but there was some evidence that increasing age led to increased rate of mortality.

By definition, dialysis dependent patients have evidence of organ failure. They are typically older with higher clinical frailty scores and co-morbidities such as hypertension, ischemic heart disease and diabetes mellitus making them vulnerable to COVID-19 as demonstrated by our cohort. These patients are often not considered suitable for intensive care management and as a result are treated at a ward-based level. Despite the limitations as described above, we believe this study provides impetus for further work clarifying the role of CPAP in treating COVID-19 dialysis dependent patients. Furthermore, this study shows that with education and a multi-disciplinary approach, CPAP can be successfully delivered outside the usual setting to not only relieve pressures on ICU capacity but allow simultaneous dialysis on non-respiratory wards.

## Supplementary Information

**Additional file 1.**

## Data Availability

The data analysed for this study is available from the corresponding author on request.

## Abbreviations

CPAP: Continuous Positive Airway Pressure
ICU: Intensive Care Units
RRT: Renal Replacement Therapy
HHD: Home Haemodialysis
ICHD: In-centre Haemodialysis
PD: Peritoneal Dialysis
CSF: Clinical Frailty Scores
IQR: Interquartile Range
PEEP: Positive End Expiratory Pressure
ARDS: Acute Respiratory Distress Syndrome
MOF: Multi-organ Failure
CVVH: Continuous Veno-Venous Haemofiltration
HDF: Hemodiafiltration
LMWH: Low Molecular Weight Heparin
