# Correction to: Using CPAP in COVID-19 patients outside of the intensive care setting: a comparison of survival and outcomes between dialysis and non-dialysis dependent patients

**DOI:** 10.1186/s12882-021-02382-2

**Published:** 2021-05-10

**Authors:** Lauren Floyd, Madelena Stauss, Joshua Storrar, Parthvi Vanalia, Anna France, Ajay Dhaygude

**Affiliations:** 1 Department of Nephrology, Royal Preston Hospital, Lancashire Teaching Hospitals NHS Foundation Trust, Preston, UK; 2 University of Central Lancashire, Lancashire, UK

**Correction to: BMC Nephrol 22, 144 (2021)**

**https://doi.org/10.1186/s12882-021-02341-x**

Following publication of the original article [1], the authors informed us that accidently omitted to add the acknowledgements to NIHR Lancashire Clinical Research Facility. The Acknowledgements has been updated as follows:
